# Supplementary material for: Superior colliculus saccade motor bursts do not dictate movement kinematics
Source: Commun Biol. 2022 Nov 11;5:1222. doi: 10.1038/s42003-022-04203-0 (PMC9652463; doi:10.1038/s42003-022-04203-0)
Supplement: Supplementary file 2 — Description of Additional Supplementary Files [file 42003_2022_4203_MOESM2_ESM.pdf]

Description of Additional Supplementary Files

File name: Supplementary Data 1

Description: The source data behind Figs. 1-5 of the paper

File name: Supplementary Data 2

Description: The source data behind Figs. 6-8 of the paper
