# Supplementary material for: Superior colliculus saccade motor bursts do not dictate movement kinematics
Source: Commun Biol. 2022 Nov 11;5:1222. doi: 10.1038/s42003-022-04203-0 (PMC9652463; doi:10.1038/s42003-022-04203-0)
Supplement: Supplementary file 5 — Reporting Summary [file 42003_2022_4203_MOESM5_ESM.pdf]

nature portfolio

Corresponding author(s): Hafed

Last updated by author(s): August 15, 2022

Reporting Summary

Nature Portfolio wishes to improve the reproducibility of the work that we publish. This form provides structure for consistency and transparency in reporting. For further information on Nature Portfolio policies, see our Editorial Policies and the Editorial Policy Checklist.

Statistics

For all statistical analyses, confirm that the following items are present in the figure legend, table legend, main text, or Methods section.

n/a	Confirmed	
☐	☒	The exact sample size (*n*) for each experimental group/condition, given as a discrete number and unit of measurement
☐	☒	A statement on whether measurements were taken from distinct samples or whether the same sample was measured repeatedly
☐	☒	The statistical test(s) used AND whether they are one- or two-sided<br>*Only common tests should be described solely by name; describe more complex techniques in the Methods section.*
☐	☒	A description of all covariates tested
☐	☒	A description of any assumptions or corrections, such as tests of normality and adjustment for multiple comparisons
☐	☒	A full description of the statistical parameters including central tendency (e.g. means) or other basic estimates (e.g. regression coefficient) AND variation (e.g. standard deviation) or associated estimates of uncertainty (e.g. confidence intervals)
☐	☒	For null hypothesis testing, the test statistic (e.g. *F*, *t*, *r*) with confidence intervals, effect sizes, degrees of freedom and *P* value noted<br>*Give P values as exact values whenever suitable.*
☒	☐	For Bayesian analysis, information on the choice of priors and Markov chain Monte Carlo settings
☒	☐	For hierarchical and complex designs, identification of the appropriate level for tests and full reporting of outcomes
☐	☒	Estimates of effect sizes (e.g. Cohen's *d*, Pearson's *r*), indicating how they were calculated

Our web collection on statistics for biologists contains articles on many of the points above.

Software and code

Policy information about availability of computer code

Data collection	Signals were digitized using the Plexon MAP (Multi-channel acquisition processor).
Data analysis	Matlab 2014, 2022

For manuscripts utilizing custom algorithms or software that are central to the research but not yet described in published literature, software must be made available to editors and reviewers. We strongly encourage code deposition in a community repository (e.g. GitHub). See the Nature Portfolio guidelines for submitting code & software for further information.

Data

Policy information about availability of data

All manuscripts must include a data availability statement. This statement should provide the following information, where applicable:

- Accession codes, unique identifiers, or web links for publicly available datasets
- A description of any restrictions on data availability
- For clinical datasets or third party data, please ensure that the statement adheres to our policy

The datasets generated during and/or analysed during the current study are available from the corresponding author on reasonable request.

Field-specific reporting

Please select the one below that is the best fit for your research. If you are not sure, read the appropriate sections before making your selection.

☒ Life sciences ☐ Behavioural & social sciences ☐ Ecological, evolutionary & environmental sciences

For a reference copy of the document with all sections, see nature.com/documents/nr-reporting-summary-flat.pdf

Life sciences study design

All studies must disclose on these points even when the disclosure is negative.

Sample size	We used four monkeys in total. This is higher than the usual 2, viewed as the best compromise between replicability of results and minimization of the use of research animals. In some analyses (e.g. Fig. 1), the numbers of neurons were constrained by the need to control for factors like depth from the SC surface. In others, the numbers of neurons were constrained by whether we had enough trial repetitions (e.g. Fig. 8) For behavioral data, we varied eccentricities and directions from the horizontal meridian with multiple repetitions per analyzed bin.
Data exclusions	No data were excluded. Some analyses required constraining the choice of neurons or movements to a given bin range, but this was explained (e.g. to control for possible confounding factors like depth from the SC surface or violations of vector-matching in the experiments of Figs. 6-8).
Replication	We replicated results from four monkeys. Also, the results are consistent with other evidence from the published literature (when viewed in the light of the current study). Therefore, four animals is a reasonable compromise given the need to reduce the overall number of animals used in research.
Randomization	We did not randomize the animals or neurons into groups. We just classified neurons or saccades based on visual field location and/or depth from the SC surface. However, in offline sorting, we were unbiased in the selection of neurons to include into analysis.
Blinding	Not blinded.

Reporting for specific materials, systems and methods

We require information from authors about some types of materials, experimental systems and methods used in many studies. Here, indicate whether each material, system or method listed is relevant to your study. If you are not sure if a list item applies to your research, read the appropriate section before selecting a response.

Materials & experimental systems

n/a	Involved in the study
☒	☐ Antibodies
☒	☐ Eukaryotic cell lines
☒	☐ Palaeontology and archaeology
☐	☒ Animals and other organisms
☒	☐ Human research participants
☒	☐ Clinical data
☒	☐ Dual use research of concern

Methods

n/a	Involved in the study
☒	☐ ChIP-seq
☒	☐ Flow cytometry
☒	☐ MRI-based neuroimaging

Animals and other organisms

Policy information about studies involving animals; ARRIVE guidelines recommended for reporting animal research

Laboratory animals	Male rhesus macaque monkeys (7-10 years old)
Wild animals	not applicable
Field-collected samples	not applicable
Ethics oversight	Regierungspräsidium Tübingen

Note that full information on the approval of the study protocol must also be provided in the manuscript.
